# In Vitro Hair Growth Promoting Effect of a Noncrosslinked Hyaluronic Acid in Human Dermal Papilla Cells

**DOI:** 10.1155/2021/5598110

**Published:** 2021-10-31

**Authors:** Nicola Zerbinati, Sabrina Sommatis, Cristina Maccario, Maria C. Capillo, Serena Di Francesco, Raffaele Rauso, Marina Protasoni, Edoardo D'Este, Daniela Dalla Gasperina, Roberto Mocchi

**Affiliations:** ^1^Department of Medicine and Surgery, University of Insubria, Varese 21100, Italy; ^2^UB-CARE S.r.l., Spin-off University of Pavia, Pavia 27100, Italy; ^3^Department of Maxillofacial Surgery, University of Campania “Luigi Vanvitelli”, Caserta 81100, Italy; ^4^Department of Dermatology, Centro Medico Polispecialistico, Pavia 27100, Italy

## Abstract

Dermal papilla cells (DPCs) are a source of nutrients and growth factors, which support the proliferation and growth of keratinocytes as well as promoting the induction of new hair follicles and maintenance of hair growth. The protection from reactive oxygen species (ROS) and the promotion of angiogenesis are considered two of the basal mechanisms to preserve the growth of the hair follicle. In this study, a noncrosslinked hyaluronic acid (HA) filler (HYDRO DELUXE BIO, Matex Lab S.p.A.) containing several amino acids was tested with in vitro assays on human follicle dermal papilla cells (HFDPCs). The experiments were carried out to investigate the possible protection against oxidative stress and the ability to increase the vascular endothelial growth factor (VEGF) release. The results demonstrated the restoration of cell viability against UVB-induced cytotoxicity and an increase in the VEGF secretion. These data demonstrate the capability of the product to modulate human dermal papilla cells, suggesting a future use in mesotherapy, a minimally invasive local intradermal therapy (LIT), after further clinical investigations.

## 1. Introduction

Hair is a specialized derivative structure of the skin and a defining characteristic of the human integumentary system. Hair originates from a follicular structure, consisting of epithelial components (matrix and outer-root sheath) and dermal components (dermal papilla and connective tissue sheath). In particular, dermal papilla cells (DPCs), located at the base of the hair follicle, are a specialized mesenchymal component of the hair that are very important in the morphogenesis and regeneration of hair growth. They act as a source of nutrients and growth factors, which support the proliferation and growth of keratinocytes as well as promoting the induction of new hair follicles and maintenance of hair growth [1]. The hair follicle cycle includes a rapid phase of growth of the hair sheat (anagen phase), followed by a transitionary regression (catagen) and quiescence (telogen) phase, and it is strictly regulated by different growth factors [2]. Among these, the most involved in the regulation of cell proliferation, differentiation, and survival is insulin-like growth factor-I(IGF-I), tumor growth factor-*β* (TGF-*β*), and vascular endothelial growth factor (VEFG) [3–6]. IGF-I has been shown to be one of the primary regulators in vitro able to suppress premature entry of follicles in the regression phase [7]. TGF-*β*1, one of the three isoforms of TGF-*β*, is known to induce catagen progression in mouse hair growth and to inhibit the in vitro growth of human keratinocytes [8, 9]. VEGF is a crucial regulator of physiological angiogenesis during skin development, and its overexpression in hair follicle epithelial cells is responsible for perifollicular vascularization and acceleration of hair regrowth in mice [10]. Dysregulation of these growth factors together with the alteration of the hair cell cycle is the trigger factor of one of the most common hair disorders, alopecia. Alopecia is a complex phenomenon where different factors, as genetic, ageing, infections, stress, and hormone imbalances, are involved with a strong social impact [1]. Only two drugs, minoxidil and finasteride, have been approved by the US Food and Drug Administration (FDA) [11], but the scientific research is in continuous development to introduce new hair growth-stimulating active compounds. Therefore, preclinical screening models have a primary role to assess their efficacy. The culture of microdissected hair follicles (HFs) and scalp skin is the best choice, permitting to have the same shaft growth that seen in vivo [12]. However, there are some limitations related to the method as the invasive procedure, the limited number of follicles extracted, and the short time to be cultured and maintained ex vivo. As recently summarized by Znidaric and his team, monolayer cultures of specific cell types with a role in hair follicle morphogenesis and hair growth cycles, as dermal papilla cells (DPCs), can be used as a valid alternative [13]. DPCs were selected to demonstrate the hair growth modulating effects of several growth factors, cytokines, and herbal products, analyzing different key biomarkers of cell proliferation, apoptosis, and analysis of growth factors. The obtained results were further confirmed with in vivo and ex vivo studies [14]. In our article, a noncrosslinked HA basal structure with a wide range of amino acids (lysine, proline, leucine, isoleucine, valine, serine, and alanine), whose main property is to stimulate anabolic functions of scalp cells such as replication, protein synthesis, and production of extracellular matrix components and promoting the growth of hair follicles [15, 16], was investigated using human DPCs. The experiments were carried out focusing on the possible protective effect in response to stress-inducing conditions triggered by environmental damage, as the oxidative stress related to ultraviolet (UV) radiation and the ability to promote VEGF release. Our results consolidate the data recently published on human keratinocytes [17], giving the product a scientific support to be a good candidate for biorevitalization of the hair follicle and future clinical investigations.

## 2. Materials and Methods

### 2.1. Cell Culture and Sample Preparation

Human follicle dermal papilla cells (HFDPCs) were purchased by PromoCell. The cell line was cultured in a specific medium (follicle dermal papilla cell growth medium-ready to use with its supplement mix, PromoCell) in conditions of complete sterility and maintained at 37 °C with 5% carbon dioxide (CO_2_) atmosphere. The product HYDRO DELUXE BIO was weighed and dissolved at the concentration of 80 mg/ml in a complete cell culture medium.

### 2.2. Cytotoxicity Assay (MTT Test)

MTT (3-[4,5-dimethylthiazol-2-yl]-2,5 diphenyl tetrazolium bromide), a colorimetric assay, is used to test in vitro cytotoxic effects and cell proliferation [18]. Briefly, cells were homogeneously seeded in 96-well plates at the density of 1.7 × 10^4^ and incubated at 37 °C, with 5% CO_2_ in a humidified atmosphere. After 24 h, cells were treated with scalar dilutions (1 : 2) of the product, starting from 80 mg/ml in complete medium. Untreated cells were used as control. At the end of treatment, cells were incubated with the MTT (Sigma-Aldrich) solution (1 mg/ml) at 37 °C for 2 h. Subsequently, the solution was removed and replaced with 100 *μ*l of dimethyl sulfoxide (DMSO). Absorbance was determined at 570 nm using a microplate reader (MultiSkan Go, Thermo Fischer Scientific). Cell viability was calculated measuring the difference in optical density (OD) of the tested product with respect to control (untreated cells), as described by the following equation (1):

(1) $$\begin{array}{c} \text{cell viability}\left(\%\right)=\left[{\text{OD}}_{\left(570\text{nm}\right)}\text{test product}/{\text{OD}}_{\left(570\text{nm}\right)}\text{ control}\right]\times 100. \end{array}$$

### 2.3. Cell Viability against Oxidative Damage

The cytoprotective effect exerted by the tested product on HFDPCs was evaluated under oxidative stress induced by UVB radiation. For the preparation of the assay, cells were homogeneously seeded in a 96-well plate at the density of 1.7 × 10^4^ and incubated at 37°C, with 5% CO_2_ in a humidified atmosphere. HFDPCs were treated with the tested product at the two non-cytotoxic concentrations (40 and 80 mg/ml) and subsequently irradiated with 10 mJ/cm^2^ UVB using a 16 W CAMAG (Muttenz, CH) UV lamp emitting at 302 nm. Untreated cells were used as control. The dose was verified using Spectroline DRC-100× digital radiometer (Spectronics Corporation). Cells were then cultured in complete medium (recovery time) or treated again with the product for a further 24 h to evaluate a possible protective effect against UVB radiation. Survival of cells was monitored by the MTT assay.

### 2.4. VEGF Estimation

The proangiogenic efficacy was determined by measuring the VEGF levels into extracellular medium by an ELISA kit (Quantikine Human ELISA assay kit, R&D Systems). To perform the test, cells were seeded in a 6-well plate and treated with the tested product at the two non-cytotoxic concentrations (40 and 80 mg/ml) verified by the MTT test in a complete medium with low fetal bovine serum (FBS, Gibco-Fischer Scientific) concentration (1%). At the end of the treatment, supernatants were collected, and the ELISA assay was performed following the manufacturer's instructions. Human VEGF standard was reconstituted and used to build the standard curve (31.25-1000 pg/ml). Standards and samples were added in duplicate into wells, and the VEGF amount was measured using 4PL curve considered the best-fit line. VEGF increase was calculated with the following equation (2):

(2) $$\begin{array}{c} \%\text{increase in VEGF}=\left[\left(\text{C}-\text{D}\right)/\text{D}\right]\times 100. \end{array}$$

where *C* is the concentration of VEGF (pg/ml) in cells treated with the tested product, and *D* is the concentration of VEGF (pg/ml) in untreated cells.

### 2.5. Statistical Analysis

Results are presented as mean ± standard deviation (SD) of at least three independent experiments performed in duplicate. Statistical significance was calculated in all experimental sets using the one-way ANOVA with Dunnett's multiple comparison posttest. *p* < 0.05 was considered to indicate a statistically significant difference compared to the relative controls. Statistical analysis was performed using GraphPad Prism version 9.0.0 (GraphPad Software, Inc).

## 3. Results

### 3.1. Evaluation of the Cytotoxicity by the MTT Assay

**Figure 1:**
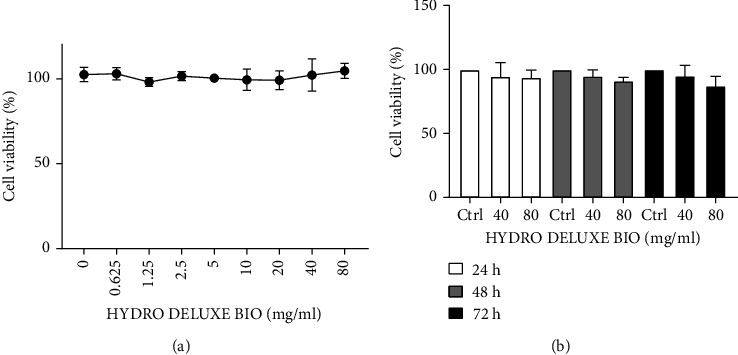
(a) Cell viability of HFDPCs cultured in vitro after treatment with HYDRO DELUXE BIO evaluated using the MTT assay. (b) HFDPCs treated with 40 and 80 mg/ml for 24, 48, and 72 h compared to untreated cells (*n* = 3; replicates = 3).

The cytotoxicity test is required to evaluate the effect of the tested product on cell viability and to identify the appropriate concentrations that do not cause a decrease in cell respiration exceeding 20%. In Figure 1(a), the cell viability after 24 h treatment of HFDPCs cells with different concentrations of the tested product is shown, expressed as a percentage compared to the control (untreated cells). The tested product did not show cytotoxic activity at all the tested concentrations. The same result was obtained dissolving the product in a medium with low concentration (1%) of FBS (data not shown). 40 and 80 mg/ml concentrations were also tested for long-time treatment (Figure 1(b)), demonstrating the absence of cytotoxicity up to 72 h and therefore used for the subsequent experiments.

### 3.2. Protection against Oxidative Damage

**Figure 2:**
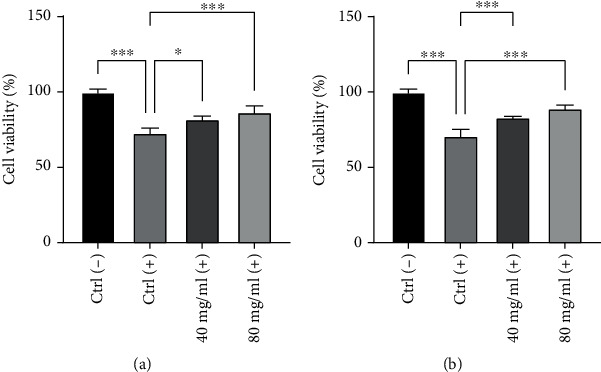
Protective effect of HYDRO DELUXE BIO against UVB-induced oxidative damage. HFDPCs were treated with 40 and 80 mg/ml and irradiated with UVB. MTT was performed after 24 h in complete medium post-irradiation (a) or further 24 h treatment post-irradiation (b). Ctrl (-): untreated cells; Ctrl (+): untreated and UVB-irradiated cells; 40 mg/ml (+) and 80 mg/ml (+): treated and UVB-irradiated cells. Values of ^∗^*p* < 0.05 and ^∗∗∗^*p* < 0.001 were considered statistically significant compared to untreated cells and untreated UVB-irradiated cells (*n* = 3; replicates =4).

Oxidative stress from UV radiation has been identified as an important etiological factor for the induction of cell senescence and cell death. Moreover, in the last years, it has been highlighted the oxidative stress and consequently the increased levels of reactive oxygen species (ROS) as a possible cause of the progression and onset of hair loss [19, 20]. UVB was then used as a stressor to induce cytotoxicity. HFDPCs were irradiated with different doses of UVB (range 2.5–20 mJ/cm^2^) to identify a decrease in cell respiration (index of cell viability) between 25% and 30%. From the obtained results, 10 mJ/cm^2^ dose was chosen for the subsequent experiments (data not shown). Therefore, after 24 h treatment with the highest non-cytotoxic concentrations of 40 and 80 mg/ml, HFDPCs were irradiated with 10 mJ/cm^2^ UVB dose and cultured for further 24 h in complete medium or in presence of the tested product.  Figure 2 and Table 1 show the protection against oxidative damage after treatment with 40 and 80 mg/ml of the tested product in UVB-irradiated cells after 24 h of recovery in complete medium (Figure 2(a)) and further 24 h in medium added with the tested compound (Figure 2(b)). Untreated and non-irradiated control cells were also reported. HFDPCs that have undergone treatment with the product, irradiated and then left in their complete medium for recovery, showed a percentage of viability higher than untreated UVB-irradiated cells. The observed increase is about 12% and 19% with the tested product at 40 and 80 mg/ml, respectively. Similar data were obtained after a second treatment after irradiation, with an increase of cell viability of about 17% and 25% after treatment with 40 and 80 mg/ml, respectively. All results with different times of treatments showed significant protection against UVB at the two concentrations tested compared to UVB-irradiated cells.

### 3.3. Evaluation of VEGF Release

**Figure 3:**
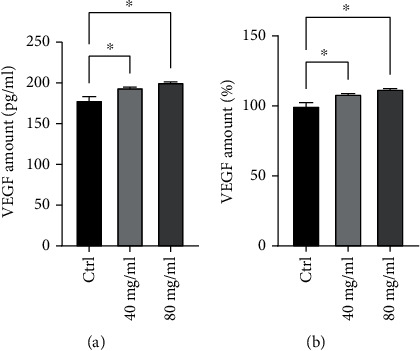
Effect of 24 h treatment with HYDRO DELUXE BIO (40 and 80 mg/ml) on VEGF secretion expressed in pg/ml (a) and percentage (b) compared to untreated cells (Ctrl). Values of ^∗^*p* < 0.05 were considered statistically significant compared to untreated cells (*n* = 3; replicates = 2).

VEGF is known to play an important role in mediating angiogenesis during the hair growth cycle. VEGF secretion is expressed as concentration in pg/ml and percentage in cells treated with the formulation at the concentrations of 40 and 80 mg/ml compared to untreated cells (Figure 3 and Table 2). Treatment of HFDPCs with the product increased VEGF secretion. In particular, the increase of VEGF measured resulted to be statistically significant (*p* < 0.05) for both the tested concentrations and equal to 9% and 12% after treatment with 40 and 80 mg/ml, respectively.

## 4. Discussion

Hair loss (alopecia) is a common disorder due either to a failure to regrow hair fibers from existing hair follicles, to extrafollicular environmental factors that affect follicular stem cell activity, or to the loss of hair follicles themselves [21]. The causes can be multiple and related to intrinsic and extrinsic factors. Intrinsic factors are related to individual genetic and epigenetic mechanisms with interindividual variation, as familiar premature greying and androgenetic alopecia (AGA). Extrinsic factors include UV radiation, smoking, and nutrition. Furthermore, the negative social aspect related to hair loss should not be underestimated [22]. There is still an unmet need for therapies capable to provide satisfying and long-term results. Recently, Alsalhi and colleagues have been published an interesting review related to the novel drug delivery approaches for the management of hair loss, highlighting the use of the mesotherapy, a minimally invasive and relatively painless procedure [23]. This technique includes injections of medications and/or vitamin mixture directly into the skin (dermis or subcutaneous layer) for the treatment of lipolysis, skin rejuvenation, pigmentation, and as already mentioned, hair loss. In this field, mesotherapy works to improve hair quality, moisture, microcirculation, and nutrient supply, prolongs the anagen phase, and inhibits 5-*α* reductase, a converting enzyme for androgenic hormones. Nevertheless, its efficacy is still controversial and conditioned by the administration's techniques and the right choice of ingredients, like minoxidil, finasteride, hyaluronic acid and amino acids [24]. Specifically, HA injectable products are used to promote skin rejuvenation by increasing the density of dermal collagen fibers produced by activated fibroblasts, thereby improving skin hydration and firmness. All these characteristics can be demonstrated through clinical instrumental evaluation on human volunteers [25]. However, showing the same claim does not mean having the same cellular response, as demonstrated by a study conducted by Jager and colleagues, in which five injectable HA products used in mesotherapy elicited divergent physiological processes in human skin fibroblast in vitro [26]. Another article focused on in vitro study has been published by Deglesne [27], showing the effect of an HA injectable product to promote human skin fibroblast viability and elastin and procollagen type I production. Based on these articles, we wanted to investigate an HA injectable product with a potential efficacy in the biorevitalitation of the hair follicle. The tested product is characterized by a noncrosslinked HA added with several amino acids, as lysine, proline, leucine, isoleucine, valine, serine, and alanine. As recently published [17], the product shows high biocompatibility and the ability to significantly reduce the expression of the inflammatory marker IL-8 and the vascular growth factor in human keratinocytes. Considering the constant interaction between keratinocytes and dermal papilla cells in the hair structure, our experiments were carried out using human follicle dermal papilla cells (HFDPCs). Owing to their role in follicular morphogenesis and hair growth cycle, this cell line represents an excellent choice among the in vitro models to study innovative hair growth-promoting agents [14]. We investigated the effect of the new enriched formulation on hair growth through the protective effect against oxidative stress induced by UVB radiation and the expression of VEGF levels in HFDPCs. A preliminary MTT test was performed to detect the right concentrations of the formulation and UVB dosage to be used for further tests. The results demonstrated a good biocompatibility of the product, choosing the non-cytotoxic concentrations of 40 and 80 mg/ml for the tested product and 10 mJ/cm^2^ dose of UVB radiation. Our results highlighted the restoration of cell viability against UVB-induced cytotoxicity and a significant increase in the VEGF expression. Taken all together, the data suggest that the product modulates human keratinocytes and dermal papilla cells, two of the main cell population of hair, suggesting a positive in vitro effect on hair growth.

## 5. Conclusions

Overall, the results of this study demonstrated that HYDRO DELUXE BIO (Matex Lab SpA) has a dual action in the experimental conditions tested: it can protect dermal papilla cells from oxidative stress and increase VEGF levels. Further in vivo experiments will be necessary with the main purpose to introduce the product in the field of mesotherapy, a minimally invasive technique characterized by the use of limited intradermal injections in the target tissue to exploit low doses of the product for medical and cosmetic purposes.

## Figures and Tables

**Table 1:** Percentage changes (mean ± SD) in cell viability after 24 h of treatment with HYDRO DELUXE BIO at the two non-cytotoxic concentrations of 40 and 80 mg/ml and UVB-irradiation. MTT test was performed after 24 h post-irradiation.

Sample	24 h recovery Post-irradiation	24 h treatment Post-irradiation
Ctrl (-)	100 ± 3.14	100 ± 3.08
Ctrl (+)	72.98 ± 4.39	70.90 ± 5.58
HYDRO DELUXE BIO, 40 mg/ml (+)	81.96 ± 3.22	83.16 ± 1.90
HYDRO DELUXE BIO, 80 mg/ml (+)	86.71 ± 5.40	89.16 ± 3.25

**Table 2:** Values of VEGF levels, expressed as concentration and percentage, in HFDPCs after treatment with the tested product HYDRO DELUXE BIO (40 and 80 mg/ml).

Sample	VEGF levels (pg/ml)	VEGF levels (%)
Ctrl	179.13 ± 5.97	100 ± 3.3
HYDRO DELUXE BIO, 40 mg/ml	194.48 ± 2.25	108.57 ± 1.26
HYDRO DELUXE BIO, 80 mg/ml	200.87 ± 2.26	112.14 ± 1.26

## Data Availability

Data are included in the text; raw data are available by corresponding author.
